# The perspectives of politicians on tobacco control in Turkey

**DOI:** 10.1093/eurpub/cky152

**Published:** 2018-10-29

**Authors:** Hilal Ozcebe, Toker Erguder, Mehmet Balcilar, Pavel Ursu, Aaron Reeves, David Stuckler, Andrew Snell, Gauden Galea, Bente Mikkelsen, Kristina Mauer-Stender

**Affiliations:** 1 Department of Public Health, Faculty of Medicine, University of Hacettepe, Ankara, Turkey; 2 World Health Organisation Country Office, Ankara, Turkey; 3 Eastern Mediterranean University, Famagusta, Turkey; 4 Montpellier Business School, Montpellier, France; 5 University of Pretoria, Pretoria, South Africa; 6 Department of Social Policy and Intervention, University of Oxford, Oxford, England; 7 Department of Social and Political Sciences, Università Bocconi, Milan, Italy; 8 World Health Organisation Regional Office for Europe, Copenhagen, Denmark; 9 World Health Organisation Country Office, Beijing, China

## Abstract

**Background:**

Tobacco use is a leading but preventable cause of non-communicable diseases and premature death. The legislature has a key role in setting tobacco control policies. Smoking trends are decreasing thanks to the introduction of effective tobacco control policies in Turkey and these policies may have been shaped by how politicians’ interpreted social problems that were prominent during the development and implementation of tobacco regulations.

**Aim:**

This paper explores the long-term national relationship between tobacco consumption, tobacco control policies and the associated political discourse in Turkey, considering the varying influences through national leadership on this important public health agenda. This relationship is studied by comparing a time series analysis of tobacco consumption trends with a policy analysis of the minutes of deliberations at the Grand National Assembly of Turkey (GNAT).

**Methods:**

This study uses Bayesian time series analysis in order investigate whether the tobacco control policies and related activities influenced the annual per adult cigarette consumption in Turkey. We used a novel method to identify change points in tobacco trends and whether they correspond with key policy changes intended to alter usage after adjusting for the effect of other non-policy related covariates, such as the purchasing power. The policy analysis included an examination of the minutes of deliberations at the GNAT—which is the Turkish parliament and unicameral Turkish legislature—1 year before and 1 year after the break years associated with an increase or decrease in tobacco consumption.

**Results and recommendations:**

Tobacco consumption increased with the encouragement of tobacco production and the entrance of multinational companies in the country in 1976 and 1993, respectively. The National Tobacco Law of 1996 and comprehensive amendments in 2008, including smoke-free public places and tax increases, appear to have helped reduce tobacco consumption in Turkey. The focus of Parliamentary discussions throughout this period changed, becoming less supportive of tobacco over time. However, throughout the period there remained discussions focussing on concerns around the implications for the economy and the privatization agenda, national agriculture and the welfare of farmers. Effective control appears to require certain political ingredients to be implemented: politicians who are well informed on tobacco control measures and understand the range of issues surrounding the policies (not only those directly health-related); and supportive public health information in the community. Evidence-based public health policy should be introduced to the politicians.

## Introduction

The World Health Organization (WHO) Framework Convention on Tobacco Control (FCTC) includes interventions for successful tobacco control at the national and international level.^1^ The first step of the commitment to implement the recommended intervention is for countries to ratify the Convention. Countries then need to develop policy and legislation implementing the approaches outlined in the FCTC. Turkey presents an interesting national case study of the varying impact of tobacco control under the FCTC. Turkey signed up to the FCTC in 2005 and as of 1 July 2015, it was the only country in the world to have accomplished all six MPOWER ‘best buy’ measures for tobacco control. Despite this, Turkey continues to have a high prevalence of tobacco use, with over 40% of men still smoking.^2^^,^^3^

Political decisions play a crucial role in the design and construction of effective tobacco control programmes, which decrease tobacco use in society.^4–6^ Political decisions can be influenced by a range of players and circumstances, including the tobacco industry and the economic situation.^7–9^ Evidence-based policymaking, seeks to making decisions according to the priority of the problem, effectiveness and cost of the decision, manpower and financial resources, is often recommended to policymakers.^10^^,^^11^ But, several barriers can stop the development of effective policy such as lack of personal contact between researchers and policymakers, the time difference between election cycles, policy processes, and research time, incuriosity of the policymakers, complex policy making systems, social psychology effects including habits, stereotypes, and cultural norms of the community, lack of transdisciplinary approach and lack of advocacy skills of public health researchers.^12^ Understanding how policymakers frame debates around public health issues can help to strengthen the roles public health professionals play in advocacy and influencing policy.^12^^,^^13^

Turkey, as a tobacco producer with a high prevalence of tobacco use, had a challenging time reducing its tobacco crop and withdrawing the state from tobacco production as part of the tobacco control programme. Eventually reforms did reduce both local production and the number of multinational companies investing in Turkey, but this led to social and economic difficulties for agricultural and factory workers. Raising the welfare level of these affected sectors and supporting alternatives has proved to be an important aspect in the pursuit of this policy, and one that is not necessarily addressed alongside the clear health benefits (box 1).

Box 1 History of tobacco control in Turkey^2^

Turkey is a tobacco-producing country, providing 1.7% of total world production. Turkey has a long history of tobacco use, which is a largely male behaviour.

The Ottomans discovered tobacco at the beginning of the 17th century, tobacco ban were introduced two times until 1631. The Ottoman Empire promoted the production and trade of tobacco and started receiving taxes from tobacco farmers in 1646. The Ottoman Empire wanted to keep tobacco production and trade under his control, but was forced to give it up because of economic problems. The Reji Company, which was founded by the French, then took possession of tobacco production and trade in the country and started to give a share to the Ottoman Empire. Tobacco factories were established under the control of Reji. The Turkish tobacco was the most preferred tobacco in European countries in these years.

After establishment of Turkish Republic, the Reji company was repaid and all rights and obligations of the Company were transferred to the government control in 1925. Tobacco production, cigarette manufacturing, pricing and selling of tobacco products started to be under the control of State Monopoly (TEKEL) in 1926.

The government decided to change the national tobacco farming, production and selling policies by allowing national and international tobacco production and distribution in the country in 1979. The government allowed the international tobacco companies to import their tobacco products in 1984; to establish their own import and distribution networks in 1986, and to establish their own product facilities in 1991. The amount of tobacco imports rose and Virginia and Burley tobaccos started to be farmed instead of oriental tobacco.

A Tobacco Control Law proposal was adopted by the Grand National Assembly but it was vetoed by the president on the basis that an advertising ban was against free trade in 1992. Several organizations were working on tobacco control and they formed National Coalition on Tobacco and Health and they started to visit members of the parliament, Ministry of Health and other related governmental organizations to persuade policymakers to introduce a tobacco control law.

The Law on Preventing Harms of Tobacco Products was accepted by the Grand National Assembly, signed by the President and came into force in 1996. The major items of the Law were:

- Bans on smoking in some public places, such as health, educational and sport facilities and some government offices, and on and in public transport vehicles and premises,
- Bans on all kinds of advertising and promotion of tobacco products,
- Bans on sales of tobacco products to minors under 18 years of age,
- Inclusion of health warnings on tobacco packages and
- Instructions to all national television channels to dedicate at least 90 min per month air time to broadcasts on the harms of tobacco use.

The Tobacco and Alcohol Market Regulatory Authority (TAPDK) was established as a financially and administratively autonomous authority ‘to regulate the tobacco market, taking into the economics of the country, as well as public health concerns and also protection of social values of the community’ in 2002. TEKEL’s tobacco department was put up for privatization in 2003 and sold to British American Tobacco (BAT) in 2008.

FCTC was ratified on 28 April 2004, approved on 25 November 2004 and entered into Turkish national law. The National Action Plan was introduced to the media and community in 2007.

Law 5727 Amending the Law on Prevention of Hazards of Tobacco Control Products was accepted, and Turkey became a smoke-free country in 2008. The main items of the Law were:

- Smoking bans in all closed areas (including school premises, all hospitality workplaces and commercial taxis),
- Bans on the sale of tobacco products within schools and on their premises,
- Bans on all kinds of sponsorship in addition to the ban on advertising and promotion contained in the previous Law and
- Clearly defining the rules in cases of violation and placing the duty on the directors of the establishments to uphold the law.

This paper explores the long-term national relationship between tobacco consumption, tobacco control policies and the associated political discourse, to understand these varying influences through national leadership on this important public health agenda. We use time series analysis in order to assess the effectiveness of tobacco control interventions between 1996 and 2016 in Turkey. The Bayesian change point approach used in the study identifies trend changes in the per adult cigarette consumption, allowing us to determine whether any of these corresponds to the periods of policy interventions. The maintained hypothesis of a trend change at any point in the sample period is tested against the null hypothesis of no trend change. The identified change point corresponds to the historical events or tobacco control policy interventions given in table 1.

**Table 1:** Tobacco related historical event and tobacco control interventions during the study period

Period	Event/intervention on or immediately preceding the period
1976	Parliamentary discourses were focused on problems for tobacco farmers
1993	The law passed in 1991 allowed foreign tobacco companies to invest in Turkey and to establish their own product facilities.
	Promotion campaigns of foreign tobacco companies started after 1991.
1998	The Law on Preventing Harms of Tobacco Products (1996): Bans on smoking in some public places, such as health, educational and sport facilities and some government offices, and on and in public transport vehicles and premises, Bans on all kinds of advertising and promotion of tobacco products, Bans on sales of tobacco products to minors under 18 years of age, Inclusion of health warnings on tobacco packages and Instructions to all national television channels to dedicate at least 90 min per month air time to broadcasts on the harms of tobacco use.
2010	The Law on Prevention of Hazards of Tobacco Products (2008): Smoking bans in all closed areas (including school premises, all hospitality workplaces and commercial taxis), Bans on the sale of tobacco products within schools and on their premises, Bans on all kinds of sponsorship in addition to the ban on advertising and promotion contained in the previous Law and Clearly defines the rules in cases of violation and places the duty on the directors of the establishments to uphold the law.

## Methods

### Time series analyses

We examined long-term tobacco consumption trends in Turkey using a time series analysis to detect the change points due to tobacco control policies in the unadjusted time series data of per adult cigarette consumption. Let y_*t*_, *t* = 1, 2,…, T, denote these actual observations. The annual data cover the period from 1960 to 2016. The data are obtained from the Tobacco and Alcohol Department of the Ministry of Agriculture and Forestry. We assumed that these observations are unbiased estimates of the true mean of the tobacco consumption (*x*_t_) in a sample over our analytical period. A number of covariates such as the real purchasing power, brand substituting, promotion activities of the sellers, smuggling, etc. may affect the per adult cigarette consumption series. Our model deploys a Bayesian change point algorithm to detect incorrectly specified change points that are due to changes in these covariates. Inclusion of these variables as additional covariates in the model automatically removes their effects on the cigarette consumption, controlling for change points not due to tobacco control policies.

In the initial model, we only include first lag of the per capita (age 15+) cigarette consumption (CIG), an intercept, and a linear trend. We test for a maximum of five trend breaks in the data (the maximum number of breaks was not increased above five due to concerns about estimation precision arising from very small sample sizes. For instance, setting the maximum number of breaks equal to six may end up with sample sizes less than 10). In the test, 10% of the observations are trimmed from both ends because the tests cannot be performed at the ends of the sample due to insufficient observations.^14–16^ For the estimation, we use a Bayesian reversible jump Markov chain Monte Carlo (MCMC) sampling framework^17–19^ to evaluate the posterior model probabilities. The test found four trend breaks using 5% critical value.

### Policy analyses

The policy analysis included an examination of the minutes of deliberations at the Grand National Assembly of Turkey (GNAT)—which is the Turkish parliament and unicameral Turkish legislature—1 year before and 1 year after the break years associated with an increase or decrease in tobacco consumption. The word ‘tobacco’ was searched in GNAT minutes and the content of all speeches on tobacco were analysed. All the disclosures were transcribed and thematic analysis were conducted.^20–22^

## Results

The tobacco control policies in Turkey are examined in three sections: the first part is the period up to 1995 (preceding the first tobacco control law in Turkey); the second part includes the period from 1996 to 2008 (the years between the original tobacco control law and its amendment); the third part includes the years after 2008 (the years after the amendment of the original tobacco control legislation). The tobacco control policies in each period also reflect the changes in the structure of the government institutions, the political decisions and the implementation.

**Figure 1:**
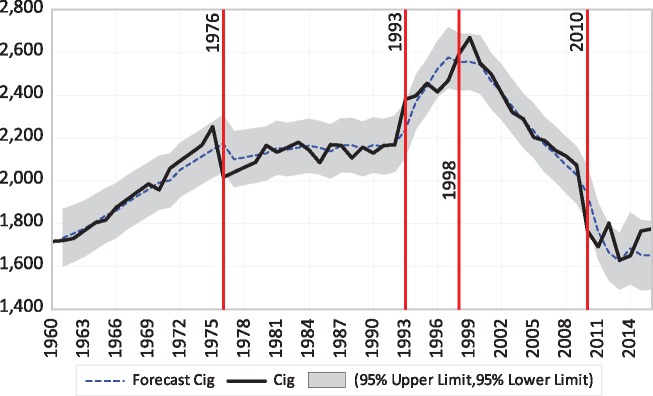
Forecasted cigarette consumption with trend breaks. The annual per adult cigarette consumption and one-step-ahead forecasts obtained from the fitted trend model with trend changes identified by the Bayesian change point algorithm is shown. Shaded region denotes 95% forecast confidence interval. Vertical straight lines are drawn at years where a significant trend change identified (see table 1)

The time series test shows four breaks happened between 1960 and 2015, in 1976, 1993, 1998 and 2010. Tobacco consumption increased until 1976 and continued increasing for the next 15 years, albeit more slowly. However, after 1993 tobacco consumption increased again but then started to decrease in 1998, a trend which accelerated after 2010. The time series is illustrated in figure 1. For the fitted model, the root mean square error for the one step forecast error is 77.84 and coefficient of determination (*R*^2^) is 0.96.

Right before the first and second marked increases in 1976 and 1993, the Parliamentary discourses were focused on problems for tobacco farmers (98.4% and 70.4% in each period). Many of interventions in these debates were in support of the Turkish tobacco industry, raising concerns about the multinational companies, smuggling, and Virginia tobacco farming, as per the example in box 2. Although the cigarette consumption was already rising before 1976, the positive increasing trend after the break in 1975 is noteworthy and policy related. Turkey was experiencing a very extreme economic depression and, faced with internal conflicts, there was shortage of all goods, and yet, despite these and other factors, cigarette consumption showed an increasing trend.

Box 2 A political disclosure example for the concern about the multinational companies, smuggling, and Virginia tobacco farming

The unfair raises in tobacco product prices cannot prevent inflation and they do not bring additional revenues to the government. On the contrary, this would aggravate increased prices, promote cigarette smuggling and thus accustom Turkish people with Virginia type tobacco.

        23 February 1975

Right before the second marked increase, in 1996, discourse on tobacco policies began to focus on issues related to tobacco control and suggested greater appetite for structural change (38.0%). The first marked decrease was seen in 1998, and the discussion topics continued to focus on these same issues, with more interventions against privatization and multinational tobacco companies (both at 12.1%). The politicians criticized privatization of tobacco, argued foreign tobacco companies were a risk to already struggling tobacco farmers, as per the example in box 3.

Box 3 A political disclosure example for the concerns about privatization of tobacco

… . .We support privatization to the extent that it improves efficiency and competitiveness. Privatization should not mean devastating local agriculture and surrendering the market to foreign monopolies. The TEKEL is not only a profitable venture but also an indispensable vehicle of our tobacco policy which is relevant to 2.5 million tobacco villagers. The aim of selling TEKEL brands and factories is to eradicate tobacco production in our country.

        7 January 1998

Before the most recent marked decrease of 2010, interventions in support of tobacco policies, structural change and control issues remained high (77.2%), but the percentage that mentioned the problems of tobacco farmers decreased to 32.9%. The focus of the debates around each break point are illustrated in table 2. Although, the cigarette consumption decreased after 2008 control policies, it rebounded after 2013. This rebound effect corresponds to the start of the Syrian civil war and could be related to the huge amount of undetected smuggling and increased cigarette consumption by Syrians which is not included in Turkish population statistics.

**Table 2:** The distribution of the tobacco related disclosures in official reports of the Turkish National Grand Assembly

Topics	First increase 1975–77 (*n* = 345 sessions)			Second increase 1992–94 (*n* = 397 sessions)			First decrease 1997–99 (*n* = 392 sessions)			Second decrease 2009–11 (*n* = 368 sessions)		
	*n*	%^a^	%^b^	*n*	%	%	*n*	%	%	*n*	%	%
Number of sessions where tobacco was mentioned	64	100.0	18.6	71	100.0	17.9	99	100.0	25.3	79	100.0	21.5
Problems of tobacco farmers^c^	63	98.4	18.3	50	70.4	12.6	65	65.7	16.6	26	32.9	7.1
Against privatization^d^	6	9.4	1.7	6	8.5	1.5	12	12.1	3.1	30	38.0	8.2
Multinational tobacco companies	3	4.7	0.9	5	7.0	1.3	12	12.1	3.1	11	13.9	3.0
Tobacco policy, structure and control issues^e^	8	12.5	2.3	27	38.0	6.8	36	36.4	9.2	61	77.2	16.6
Others^f^	4	6.3	1.2	11	15.5	2.8	13	13.1	3.3	10	12.7	2.7

a Percent of tobacco mentioned sessions.

b Percent of total sessions.

c Tobacco farmers’ problems, tobacco purchase price from farmers, reactions to limitation on farming policies, quality of Turkish tobacco and supportive policies.

d Privatization of tobacco authority, privatization of tobacco factories and the status of workers of tobacco factories.

e Tobacco policy and tobacco authority, tobacco control interventions, alternative crops, sale points, smuggling, health, prevention and education.

f Environmental issues, international relations, tobacco taxes and beneficiaries.

## Discussion

Bayesian change point analysis identified 4 trend changes in the annual per adult cigarette consumption over the period 1960–2016 in Turkey. The model controls for the effect of other covariates such as purchasing power so that the identified change points are related to exogenous events, which we interpret as tobacco control policies. The change point model has a very high ‘goodness of fit’, which is evaluated based on the one-step-ahead forecasts errors. The change point dates are 1976, 1993, 1998 and 2010. Important events before the first two periods (1976 and 1993) have resulted in faster cigarette consumption growth in Turkey. On the other, the last two change points (1998 and 2010) are preceded by significant tobacco control policies in Turkey.

Turkey was once one of the top tobacco-producing countries in the world. Its share of total world tobacco production was almost 4% before the 1990s. The Turkish Tobacco Monopoly (TEKEL) was established as a governmental institution to control the tobacco market in 1926. After 1980, TEKEL was privatized and multinational tobacco companies entered into Turkey.^2^ Granting permission for the marketing of tobacco products and allowing international companies to produce in Turkey appears to be associated with the first and second increases in tobacco use prevalence. The government started to decrease subsidies for tobacco farming and production, which led to concerns over the welfare of farmers becoming a prominent feature in Parliamentary discussions. Challenging the entrance of international companies into the Turkish tobacco market and the changing structure of tobacco policy also became prominent in the political discourse. Changing the tobacco production and marketing strategy is associated with an increase in the prevalence of smoking in the country. Politicians were more interested in tobacco farming and economic aspects of tobacco instead of health consequences of tobacco use in this period.

The first major decrease in tobacco consumption came in 1998, shortly after the first national tobacco control law of 1996. The law was the first successful implementation of tobacco control measures in the country. It completely banned tobacco advertisement and promotion, with restricted use of tobacco products in some indoor public spaces.^2^ The Parliamentary discourse before this time showed varied support of tobacco control—there was some support for policies seen to promote health, but concern around tobacco producers, including reduced tobacco farming areas, privatizing production and the need to support alternative crops. There was also an emphasis on the importance of Turkey supporting its own tobacco control policy. In this period, the topics of deliberation in the Parliament started to expand to tobacco control and health impacts.

Increases in supportive political discourse regarding tobacco control might be reflected in a broader societal shift towards anti-tobacco in this period. Besides the policies of the government, the issues raised by Members of Parliament give some idea of the various views in society, and issues raised by the electorate and constituents. However, to explore this further and to understand the interplay between effective tobacco controls, political discourse and popular opinion would require a wider discourse analysis—including media and other sources.

The second marked decrease, in 2010, came shortly after the comprehensive revision of the tobacco control law in 2008. This included implementation of a 100% smoke-free policy in all indoor public places. The original 1996 tobacco control law was expanded with the items of the MPOWER measures, promoted as priority components of FCTC or ‘best buys’. The discourse in Parliament was predominantly in support of tobacco control policies and challenging privatization of factories. The interventions mainly reflected public health approaches and debate about evidence-based policies were frequently observed in this period. This could be an effect of WHO FCTC, and also the tobacco control law and activities in the country.

Two key components seem to have enabled this most recent strengthening of tobacco control in Turkey: one was the high level of policymakers who supported a more comprehensive approach to tobacco control; the other was the associated political stability. Supportive policymakers had been in high level and relevant posts for a prolonged period leading up to this period.^2^^,^^23^

Although our study illustrates the effectiveness of tobacco control policies, we used cigarette consumption instead of the prevalence of tobacco use and our study does not uncover any health consequences. The analysis can be extended to assess the effect on quitting smoking and likely health consequences that can be observed in the long-run. Further studies are also needed to evaluate whether reduction in tobacco use is comprehensive for all age groups, all regions and sexes. We did not research tobacco related news on media, media research would be very helpful to understand the concerns of tobacco producers and factory workers in the community.

## Conclusion

A range of political and social factors influence the process of national tobacco control policy and implementation.^7^ Effective control appears to require certain political ingredients to be implemented: politicians who are well informed on tobacco control measures and understand the range of issues surrounding the policies (not only those directly health-related); and a supportive and influential public health community and provision of evidence. Evidence-based public health policy should be introduced to politicians. A public health approach in all policies will help to promote population health but advocacy should also be aware of the varied priorities that often influence the political discourse around health-related issues such as tobacco.
